# Using Nocturnal Flight Calls to Assess the Fall Migration of Warblers and Sparrows along a Coastal Ecological Barrier

**DOI:** 10.1371/journal.pone.0092218

**Published:** 2014-03-18

**Authors:** Adam D. Smith, Peter W. C. Paton, Scott R. McWilliams

**Affiliations:** Department of Natural Resources Science, University of Rhode Island, Kingston, Rhode Island, United States of America; Bowling Green State Universtiy, United States of America

## Abstract

Atmospheric conditions fundamentally influence the timing, intensity, energetics, and geography of avian migration. While radar is typically used to infer the influence of weather on the magnitude and spatiotemporal patterns of nocturnal bird migration, monitoring the flight calls produced by many bird species during nocturnal migration represents an alternative methodology and provides information regarding the species composition of nocturnal migration. We used nocturnal flight call (NFC) recordings of at least 22 migratory songbirds (14 warbler and 8 sparrow species) during fall migration from eight sites along the mainland and island coasts of Rhode Island to evaluate five hypotheses regarding NFC detections. Patterns of warbler and sparrow NFC detections largely supported our expectations in that (1) NFC detections associated positively and strongly with wind conditions that influence the intensity of coastal bird migration and negatively with regional precipitation; (2) NFCs increased during conditions with reduced visibility (e.g., high cloud cover); (3) NFCs decreased with higher wind speeds, presumably due mostly to increased ambient noise; and (4) coastal mainland sites recorded five to nine times more NFCs, on average, than coastal nearshore or offshore island sites. However, we found little evidence that (5) nightly or intra-night patterns of NFCs reflected the well-documented latitudinal patterns of migrant abundance on an offshore island. Despite some potential complications in inferring migration intensity and species composition from NFC data, the acoustic monitoring of NFCs provides a viable and complementary methodology for exploring the spatiotemporal patterns of songbird migration as well as evaluating the atmospheric conditions that shape these patterns.

## Introduction

Atmospheric dynamics fundamentally influence the timing, intensity, energetics, and geography of avian migration [1]–[3]. Wind conditions (i.e., direction and speed) around low and high pressure systems and associated frontal boundaries are particularly influential [4]. During fall migration in the northern hemisphere, many birds migrate preferentially when winds provide some tailwind component after the passage of a cold front [2,4,5; but see 6]. However, wind conditions during migratory flight can concentrate migrants at topographic barriers [7]–[9]. The increased densities of migrants at stopover sites along these ‘leading lines’ [10] can reduce energy replenishment rates via competition as well as increase the risk of predation [11]–[14]. These potential density-dependent consequences substantiate the need to identify the environmental factors that direct the movements and distribution of songbirds during migration, particularly along ecological barriers that may experience disproportionately high migrant densities.

In the northeastern United States during southbound fall migration, many nocturnal passerine migrants concentrate along the Atlantic Coast and on offshore land masses under specific weather conditions [15]–[17]. In particular, hatching-year migrants often fail to compensate for prevailing winds and are displaced to the coast or offshore (the so-called “coastal effect”; [18]), with offshore birds typically reorienting towards or along the nearest land mass near dawn [17], [19]–[21]. Although reverse migration and reorientation are common phenomena along the Atlantic Coast (e.g., [16], [17], [19], [22]), their occurrence and extent depend on a complex interplay between wind, topography, ‘on-the-ground’ distribution of resources and risk, and individual histories (e.g., [23]–[27]). The context- and weather-dependent response of nocturnal passerine migrants to coastlines, as well as their subsequent redistributional movements, implies that spatiotemporal variation in the geographic distribution of migrants aloft occurs at multiple scales along the Atlantic Coast.

The influence of weather on the magnitude and spatiotemporal patterns of nocturnal bird migration has been inferred primarily using radar (e.g., [2], [4], [28]), although the use of nocturnal flight calls (NFCs) provides a possible alternative approach. Many bird species produce distinct vocalizations during sustained flight, particularly nocturnal migration, potentially enabling the simultaneous evaluation of the magnitude, spatiotemporal patterns, and species composition of nocturnal migration [29]–[31]. In general, the temporal patterns of NFC detections associate positively with the migration intensity inferred from radar [32]–[34]. However, certain atmospheric conditions complicate the relationship between NFC detections and the number of birds aloft – NFC detections increase when visual communication is limited (e.g., low visibility and cloud ceiling, high cloud cover; summarized in [35]), but decrease with increasing ambient noise [32], [36]. Understanding these influences on the spatiotemporal patterns of NFCs will improve our ability to infer the spatial distribution and abundance of songbirds along their migration routes and ecological barriers.

We evaluated five hypotheses regarding the detection of migrant songbird NFCs along the Atlantic Coast of southern New England in relation to atmospheric and ambient conditions, as well as coastal context: (1) NFC detections vary strongly with the atmospheric conditions that influence the intensity of bird migration in general and coastal migration in particular (e.g., front passage, wind conditions, and precipitation; [2], [4]); (2) NFC detections increase under conditions expected to hinder visual communication (i.e., cloudy skies with low ceilings and reduced visibility); (3) NFC detections decrease in association with weather conditions that increase ambient noise, particularly high winds, as well as other non-wind sources of ambient noise. Finally, we expected NFC detections to vary with geographic context (i.e., relative coastal position) among sites in coastal Rhode Island. Specifically, we expected (4) more NFC detections at mainland sites relative to offshore sites, and that (5) total NFC detections and intra-night patterns of NFC detections on an offshore island would vary according to well-documented latitudinal patterns of migrant abundance on the island (i.e., migrants concentrate at the northern end of the island; [20], [37]).

## Materials and Methods

**Figure 1:**
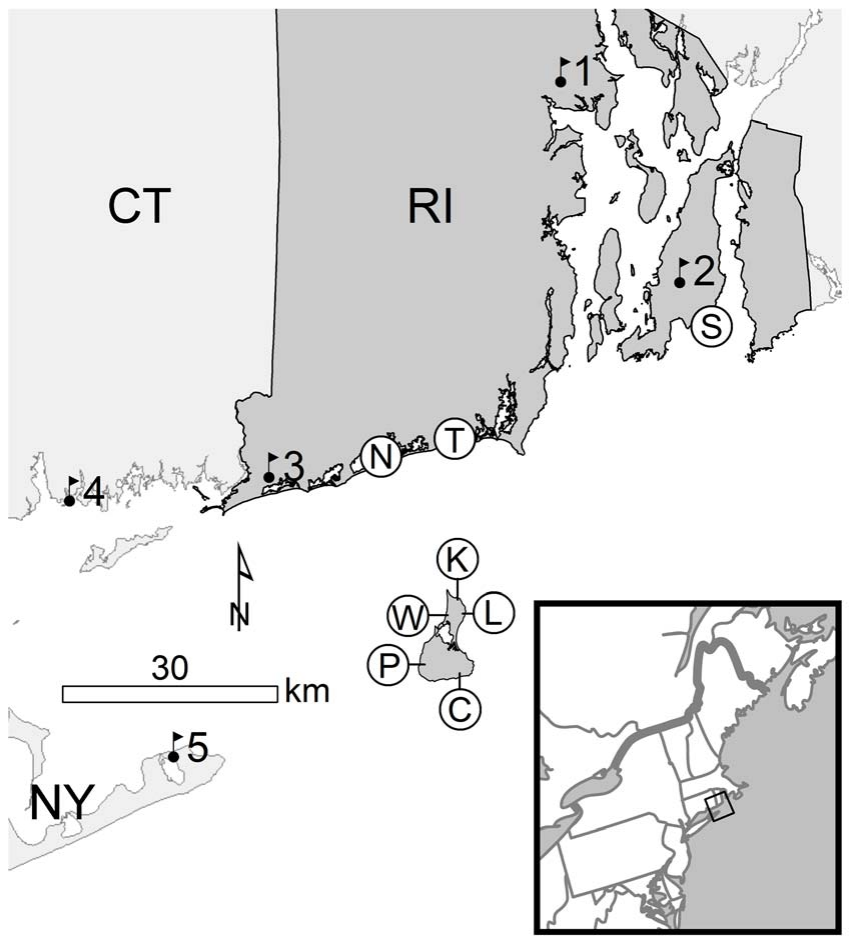
Microphone and weather station locations used to assess nocturnal flight call activity of migrating songbirds. Locations of microphones (circles) and National Weather Service ASOS stations (flags) used to examine the relationship between atmospheric conditions and the nocturnal flight call activity of migrating songbirds in southern Rhode Island (RI), Connecticut (CT), and New York (NY), USA, from September to November, 2010–2011. Microphone locations: N – Ninigret, T – Trustom, S – Sachuest, K – Kurz, W – Wash, L – Lapham, P – Pyne, and C – Comings. ASOS stations: 1 - Providence/T. F. Green State Airport, 2 - Newport State Airport, 3 - Westerly State Airport, 4 - Groton-New London Airport, and 5 - Montauk Airport. See text for more details.

During fall 2010–2011, we monitored NFC of songbirds at eight sites in southern Rhode Island, USA: two along the mainland coast (Figure 1; sites N and T), one along the southern coast of Aquidneck Island, a large (98 km^2^) nearshore island at the southern end of Narragansett Bay (Figure 1; site S), and five sites along the periphery of Block Island (25 km^2^), an offshore island located approximately 15 km south of mainland Rhode Island (Figure 1; sites C, K, L, P and W). We placed six microphones (Figure 1; sites K, L, N, S, T, and W) on protected, public lands with the authorization of the property manager; we placed the remaining two microphones (Figure 1; sites C and P) on private property with the authorization of the property owners. At each site, we recorded NFCs with a microphone (SMX-NFC; Wildlife Acoustics, Inc., Concord, MA) attached to a passive recorder (SM2BAT, 24 kHz sampling rate; Wildlife Acoustics, Inc., Concord, MA) set to maximum gain (+60 dB). The SMX-NFC possessed a relatively flat frequency response from 2–12 kHz. We mounted each microphone approximately 5–5.5 m above the ground and above the height of prevailing coastal shrub vegetation. We located monitoring sites far from artificial lighting which can disorient and concentrate nocturnal songbird migrants (reviewed in [38]), as well as anthropogenic disturbance and noise. Thus, we expected little anthropogenic influence on the calling patterns documented herein. We recorded NFCs from evening civil twilight to morning civil twilight (i.e., sun elevation approximately 6^o^ below the horizon), from 8 September to 8 November in 2010 and from 8 September to 10 November in 2011. However, we truncated the recordings 15 min prior to morning civil twilight (about 45 min prior to sunrise) due to frequent vocalizations from birds on the ground near the microphone; the resulting nightly recordings increased in length (10.3–13.0 h) over the course of the recording seasons. Occasional equipment malfunctions resulted in incomplete coverage during these periods, and we did not record at all sites in each year (Table 1).

**Table 1:** Operational summary of nocturnal flight call microphones at eight locations in southern Rhode Island, USA, during the 2010 - 2011 fall migrations.

		Mainland			Block Island				
Year		Ninigret	Trustom	Sachuest	Kurz	Wash	Lapham	Pyne	Comings
2010	Start night	-	-	14 Sep	8 Sep	11 Sep	8 Sep	9 Sep	12 Sep
	# nights operated/recorded	-	-	56/45	62/52	59/51	62/24	61/61	58/58
2011	Start night	8 Sep	8 Sep	8 Sep	9 Sep	9 Sep	9 Sep	-	9 Sep
	# nights operated/recorded	64/63	64/58	64/58	63/57	63/31	63/51	-	63/29

Discrepancies between the number of nights operated and number of nights recorded indicate that an equipment malfunction precluded recording. Monitoring ended on 8 November in 2010 and 10 November in 2011.

We filtered potential flight calls from nightly recordings using a band-limited energy detector algorithm in Raven Pro 1.3 (Build 32, Cornell Lab of Ornithology, Ithaca, NY). We specified the algorithm to extract high frequency band flight calls (i.e., within the frequency range of 6–11 kHz), which included most migratory species of warblers (Parulidae) and sparrows (Emberizidae) in eastern North America [29], [30]. We restricted our analysis to high frequency flight calls because ambient noise in the 1–5 kHz frequency range (e.g., wind, insects, or amphibians) consistently precluded the extraction of flight calls from species producing low- and mid-frequency vocalizations (e.g., thrushes, grosbeaks, tanagers). Within this 6–11 kHz frequency range, we configured the algorithm to extract potential calls 23–398 ms in duration and separated by at least 98 ms, with a signal-to-noise threshold of 3.5 dB and 30% minimum signal occupancy. We estimated the background noise against which the signal of potential calls was compared as the median power (dB) within a 12 s block with a hop size of 243 ms. We exported potential calls to individual time-stamped audio (*.wav) files. We then generated a spectrogram of each audio file using GlassOFire (www.oldbird.org) from which we manually classified calls and discarded false detections (e.g., wind, insects, rain drops, non-flight call vocalizations). To assess the effects of varying ambient noise on the detection of high frequency flight calls, we used Raven Pro to calculate the average power (dB) in the 6–11 kHz frequency range during the first hour of each night at each microphone.

We assigned NFCs to species when possible, but more commonly into a complex of similar species [30]. We further aggregated these species complexes into two families for analysis: warblers and sparrows (Table 2). While some species complexes initially contained NFCs from both families, we carefully separated presumed sparrow from warbler NFCs, typically by call length. Approximately 10% of detected flight calls were too weak to assign confidently to the level of family (9%) or belonged to other bird families (e.g., Indigo Buntings, *Passerina cyanea*; 1%); we excluded these calls from further analyses.

**Table 2:** Classification of nocturnal flight calls (NFCs) of migrating warblers (Parulidae) and sparrows (Emberizidae) recorded in southern Rhode Island, USA, during autumn in 2010 and 2011.

Classification^a^		Number of NFCs		
Group	Complex	2010	2011	Dominant constituent species^b^
Warbers	ZEEP	2,424	14,712	Blackpoll Warbler, Northern Waterthrush, Common Yellowthroat*, Magnolia Warbler; minor: Bay-breasted Warbler, Yellow Warbler, Connecticut Warbler, Chestnut-sided Warbler*, Black-and-white Warbler, Cape May Warbler; rare: Hooded Warbler, Blackburnian Warbler, Worm-eating Warbler
	1BUP	2,776	2,415	Yellow-rumped Warbler*; minor: Ovenbird, American Redstart, Black-throated Blue Warbler; rare: Blue-winged Warbler, Golden-winged Warbler
	1BDN	295	1,337	Northern Parula, Palm Warbler*; minor: Cape May Warbler, Pine Warbler*; rare: Prairie Warbler*
	NOPA	218	576	Northern Parula*
	AMRE	282	394	American Redstart
	COYE	119	421	Common Yellowthroat*
	2BUP	112	383	Yellow-rumped Warbler*, Nashville Warbler, Tennessee Warbler; minor: Black-throated Green Warbler, Mourning Warbler; rare: Orange-crowned Warbler
	BAWW	92	198	Black-and-white Warbler
	OVEN	95	137	Ovenbird
	PAWA	96	134	Palm Warbler*
	BTBW	17	47	Black-throated Blue Warbler
	CSWA	20	41	Chestnut-sided Warbler*
	NOWA	13	38	Northern Waterthrush
	MOWA	17	18	Mourning Warbler
	CAWA	9	6	Canada Warbler
	WIWA	0	10	Wilson's Warbler
Sparrows	SPAR	2,396	5,501	Chipping Sparrow, White-throated Sparrow, Song Sparrow, Savannah Sparrow; minor: Swamp Sparrow, Lincoln's Sparrow, White-crowned Sparrow; rare: Field Sparrow, Vesper Sparrow, Grasshopper Sparrow
	SAVS	716	3,111	Savannah Sparrow
	WTSP	360	1,170	White-throated Sparrow
	CHSP	351	760	Chipping Sparrow
	LISW	34	220	Swamp Sparrow; also Lincoln's Sparrow
	DEJU	94	128	Dark-eyed Junco
	FISP	3	18	Field Sparrow
	GRSP	1	13	Grasshopper Sparrow

a Classification complexes comprised of species with similar call notes, based on and modified slightly from (Evans and O'Brien 2002): ZEEP – “zeep” complex plus warbler members of the “buzz calls” complex and Cape May Warbler; 1BUP – warbler species producing single-banded calls in the “short rising seep” complex; 1BDN – warblers producing single-banded calls in the “descending seep” complex, plus Cape May Warbler; 2BUP – warbler species producing double-banded calls in the “short rising seep” complex; SPAR – sparrow members of the “descending seep,” “short rising seep,” and “buzz calls” complexes, plus long single- or double-banded sparrow calls (Chipping Sparrow, Song Sparrow, White-throated Sparrow, and Grasshopper Sparrow); LISW – sparrow members of the “buzz calls” complex.

b Scientific names: Warblers – American Redstart (*Setophaga ruticilla*), Bay-breasted Warbler (*S. castanea*), Black-and-white Warbler (*Mniotilta varia*), Blackburnian Warbler (*S. fusca*), Blackpoll Warbler (*S. striata*), Black-throated Blue Warbler (*S. caerulescens*), Black-throated Green Warbler (*S. virens*), Blue-winged Warbler (*Vermivora cyanoptera*), Canada Warbler (*Cardellina canadensis*), Cape May Warbler (*S. tigrina*), Chestnut-sided Warbler (*S. pensylvanica*), Common Yellowthroat (*Geothlypis trichas*), Connecticut Warbler (*Oporornis agilis*), Golden-winged Warbler (*V. chrysoptera*), Hooded Warbler (*S. citrina*), Magnolia Warbler (*S. magnolia*), Mourning Warbler (*G. philadelphia*), Nashville Warbler (*Oreothlypis ruficapilla*), Northern Parula (*S. americana*), Northern Waterthrush (*Parkesia noveboracensis*), Orange-crowned Warbler (*O. celata*), Ovenbird (*Seiurus aurocapilla*), Palm Warbler (*S. palmarum*), Pine Warbler (*S. pinus*), Prairie Warbler (*S. discolor*), Tennessee Warbler (*O. peregrina*), Wilson's Warbler (*C. pusilla*), Worm-eating Warbler (*Helmitheros vermivorum*), Yellow Warbler (*S. petechia*), Yellow-rumped Warbler (*S. coronata*); Sparrows – Chipping Sparrow (*Spizella passerina*), Dark-eyed Junco (*Junco hyemalis*), Field Sparrow (*Spizella pusilla*), Grasshopper Sparrow (*Ammodramus savannarum*), Lincoln's Sparrow (*Melospiza lincolnii*), Savannah Sparrow (*Passerculus sandwichensis*), Song Sparrow (*Melospiza melodia*), Swamp Sparrow (*M. georgiana*), Vesper Sparrow (*Pooecetes gramineus*), White-crowned Sparrow (*Zonotrichia leucophrys*), and White-throated Sparrow (*Z. albicollis*).

Dominant constituent species are grouped according to their expected contribution based on general impression of authors (i.e., some calls left unidentified to species were suggestive of a given species), knowledge of occurrence and migratory phenology in the region, and 5,526 banding records of relevant species from approximately 8 September to 10 November from five fall migration banding operations in southern Rhode Island in 2010–2011 (A. D. Smith unpubl. data; USFWS unpubl. data; K. Gaffett and S. Reinert unpubl. data; P. W. C. Paton unpubl. data). Species listed first are presumed to be the most common contributors; species following ‘minor' are presumed to make minor contributions; species following ‘rare’ are presumed rare contributors. Species marked with an asterisk possess flight calls that occur to some extent below 6 kHz (see text for details).

### Regional atmospheric conditions

We derived atmospheric conditions based primarily on weather data from observations at five National Weather Service Automated Surface Observing System (ASOS) stations occurring within 50 km of the centroid of microphone locations (Figure 1). ASOS reports wind speed and direction (recorded approximately 10 m above ground level), as well as precipitation amount, every minute, although the data are derived from accumulations over the previous 1 or 2 min; visibility, cloud cover, and cloud ceiling data were reported every 5 min. We calculated wind profit from wind direction and wind speed [39]; wind profit represents the distance a bird is drifted toward a specified target direction in a fixed time interval through only the effect of wind. Typically, the target direction is the migratory goal, but we specified due southeast (135^o^) as the target direction to better capture those combinations of wind direction and speed that indicate recent cold front passage and are also more likely to induce a coastal flight path, and perhaps the coastal effect [18], in migrating birds. We calculated nightly averages for weather variables from evening to morning civil twilight, thus encompassing the period of active monitoring. We also calculated the proportion of hours during a given night with at least one ASOS station reporting precipitation. Additional details of weather data acquisition and manipulation are available from the authors.

### Analysis

We used generalized additive models (GAMs; [40], [41]) to explore the association between regional atmospheric conditions on NFC detections and differences in NFC rates among sites. GAMs accommodate potential nonlinear changes in calling activity with predictor variables while allowing us to incorporate serial correlation [41]; we implemented them using the gamm function of the mgcv package [42] in R, version 2.15.2 [43]. We estimated GAMs separately for warblers and sparrows. For each group, we modeled the number of nightly NFC detections as a sum of explanatory variables using a GAM with the following formulation:
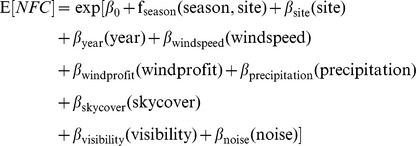
where NFC ∼ NegativeBinomial(θ), *β_0_* is an intercept, and the remaining *β*s describe their associated explanatory variables; *β_site_* and *β_year_* are categorical variables and comprise seven and two parameters, respectively. We fit a single smooth term (*f_season, site_*) to allow for potential nonlinear seasonal (i.e., day of year) effects using the default thin plate regression spline; we allowed this smooth to interact with the categorical *site* variable, resulting in the possible estimation of separate smooths for each site. We did not evaluate any additional interactions to avoid over-parameterizing the model relative to the data, nor did we expect interactions among atmospheric conditions to possess much ecological relevance. We estimated the θ parameter for the negative binomial distribution with a similarly-structured generalized linear model, although replaced the spline for seasonal effects with a third-order polynomial. A first-order autoregressive (AR-1) error structure reasonably accounted for serial correlation in residuals. We grouped the correlation structure within each site and year combination to expedite GAM estimation [41]. We centered and scaled by one standard deviation all continuous model input variables to improve estimation and facilitate the assessment of the relative importance of atmospheric conditions to NFC detections [44]; we did not modify the categorical *site* or *year* variables. We omitted cloud ceiling from consideration prior to analysis due to its high collinearity with cloud cover (i.e., variance inflation factor >10; r = −0.97, df = 124, P<0.0001). To avoid biased parameter estimates and standard errors when evaluating hypotheses, we did not eliminate any terms from the models [45]. Finally, we estimated the average seasonal discrepancy in NFC detections between mainland sites and island sites with two additional GAMs (i.e., warblers and sparrows) that dichotomized recording sites according to this geographic context.

We compared automated detections with manually quantified NFCs in a sample of 10-minute recordings to evaluate the effectiveness of our automated detector settings. We selected 12 recordings from each site, with two notable constraints: recordings contained ≥4 warbler or sparrow NFCs (median  = 7; maximum  = 45) and occurred on separate nights. We again ignored NFCs too weak to assign confidently to warblers or sparrows, or NFCs belonging to other bird families. Thus, we estimated detector efficiency under generally favorable recording conditions and compared efficiency among sites and across a restricted range of ambient noise conditions. We modeled the logit of the proportion of successfully detected NFCs in each recording (*P_detect_*) as a function of two explanatory variables, and weighted by the number of manually detected NFCs, using a generalized linear model (glm function in base R) with the following formulation:
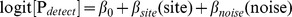
where *β_0_* is the intercept and the remaining *β*s describe their associated explanatory variables. We sampled from all eight sites, so *β_site_* comprises seven parameters. The *noise* variable represents the average power (dB) in the 6–11 kHz frequency range in each 10-minute recording.

## Results

We recorded 27,452 warbler and 14,876 sparrow flight calls in 638 microphone nights (∼7,250 h of recordings) during the fall migrations of 2010–2011 (Table 2). Most warbler NFCs (62%) were classified into a single complex (‘ZEEPs’; Table 2) dominated presumably by four species with similar flight calls; several additional species likely are represented in this complex, but to a much lesser extent. Four sparrow species were presumed responsible for nearly all (∼97%) sparrow NFCs (‘SPARs’; Table 2), although a few other species likely are represented (Table 2).

**Figure 2:**
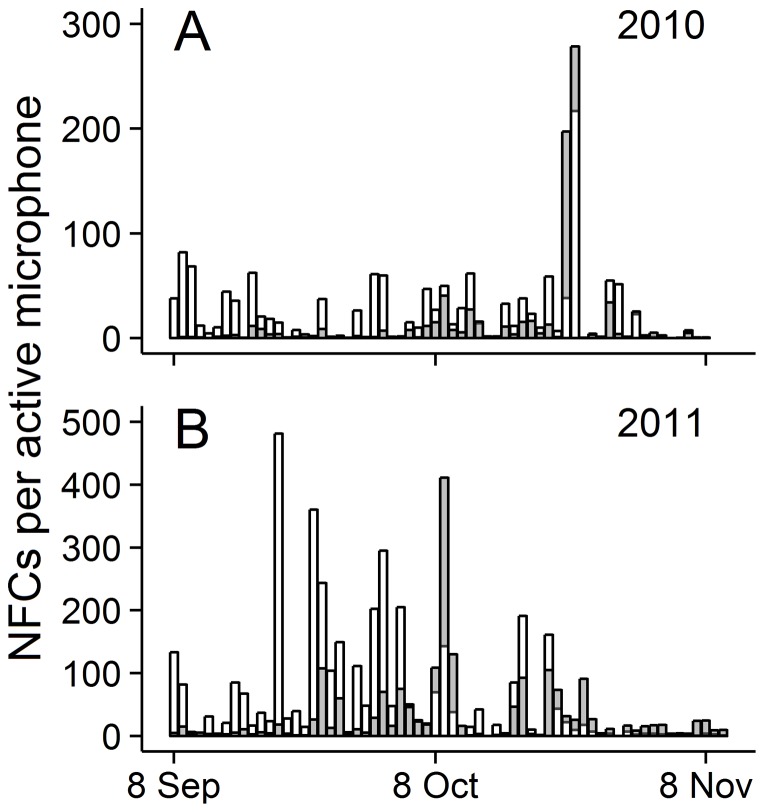
Seasonal variation in warbler and sparrow nocturnal flight call rates. Seasonal variation in the number of nocturnal flight calls (NFCs) detected per active microphone for warblers (white fill) and sparrows (gray fill) during fall migration in (A) 2010 and (B) 2011 at eight coastal sites in southern Rhode Island, USA.

**Figure 3:**
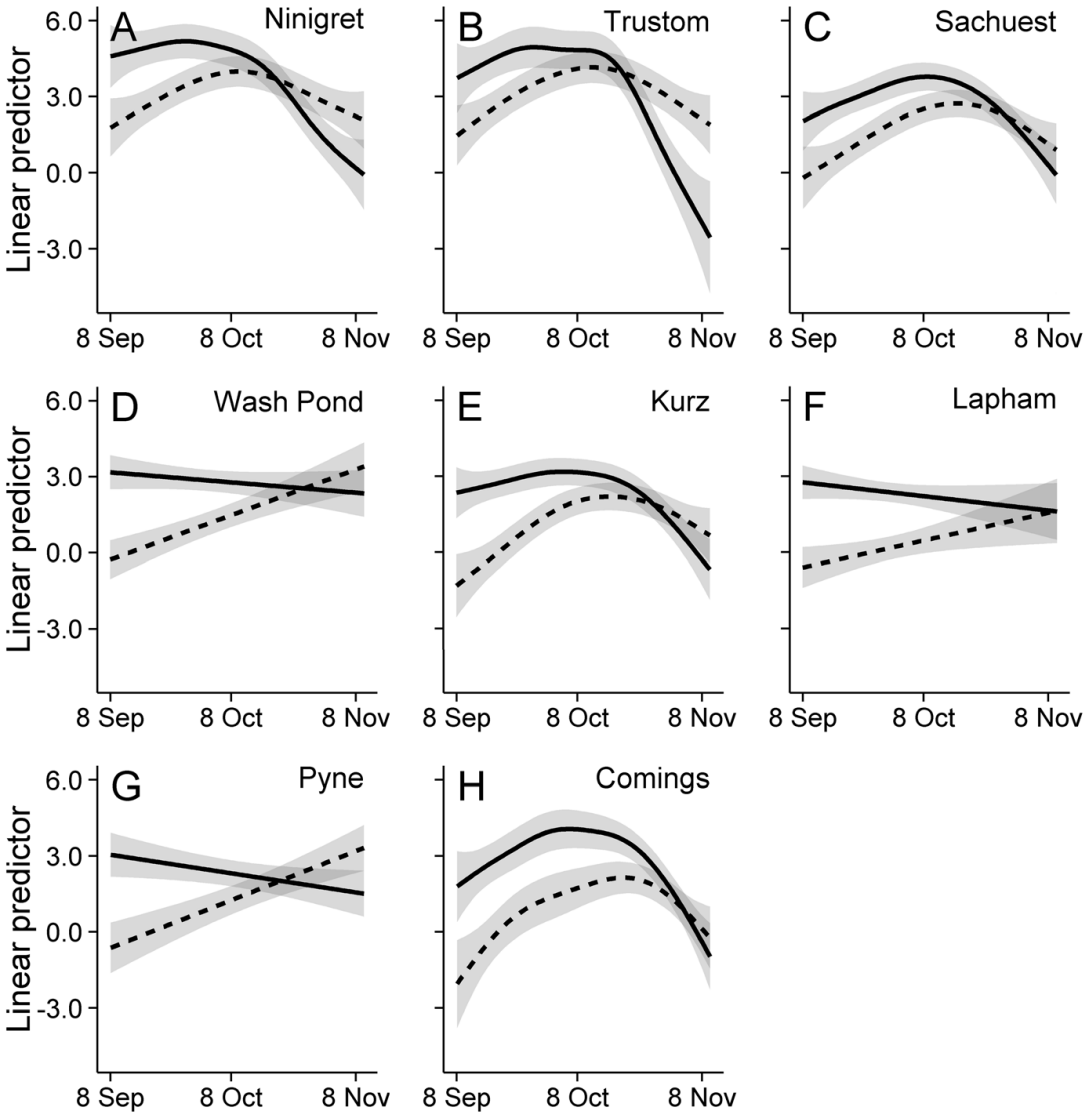
Geographic variation in the seasonal patterns of warbler and sparrow flight calls. Average seasonal pattern in warbler (solid line) and sparrow (dashed line) nocturnal flight call (NFC) detections during fall in 2010 and 2011 at eight coastal sites in southern Rhode Island, USA (see Figure 1): (A–B) two sites on the mainland coast, (C) one on Aquidneck Island, and (D–H) five on Block Island. Seasonal patterns were estimated with generalized additive models; the seasonal trend of the linear predictor (and 95% confidence interval) is illustrated with other variables held at their mean value. All plots share the same vertical scale to facilitate comparisons of NFC detections among locations. Note that each unit change in the linear predictor represents nearly a tripling of NFC detections.

Warblers and sparrows exhibited similar general patterns of NFC detections, but slightly different phenologies (Figures 2 and 3). Generally, NFC detections peaked in late September/early October (warblers) or mid-October (sparrows) and declined through the end of the season (Figure 3), regardless of site, although the data did not justify a curvilinear fit for a few sites (i.e., Figure 3D, F, G). Averaged over the entire migration period, mainland sites (Figure 3A–B) detected nearly five times the warbler NFC detections and nearly nine times the sparrow NFC detections relative to island sites (Figure 3C–H); warbler and sparrow NFC detections were similar between the single nearshore island location (Figure 3C) and Block Island locations (Figure 3D–H).

**Figure 4:**
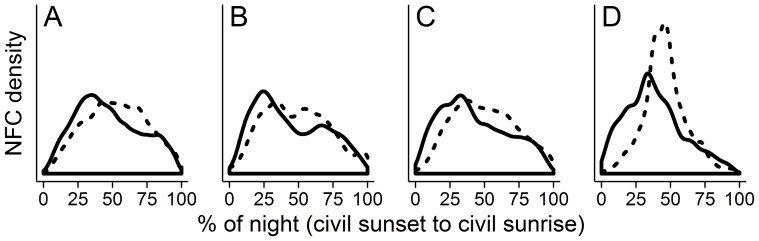
Intranight variation in warbler and sparrow nocturnal flight calls. Intranight variation in warbler (solid line) and sparrow (dashed line) nocturnal flight call (NFC) detections in 2010 and 2011 at (A) two coastal locations (sites N and T; see Figure 1) on mainland Rhode Island, (B) a single location on a nearshore island (site S), (C) three locations on northern Block Island (sites K, W, and L), and (D) two locations on southern Block Island (sites P and C). The horizontal axis uses a percentage scale to account for increasing night length throughout the study period, with 50% corresponding to approximately 2330 h EST. The vertical axis (NFC density) is identical among panels to facilitate comparisons of NFC detections; actual density values are omitted for clarity.

Warblers exhibited similar intra-night NFC detection patterns regardless of geographic context (Figure 4). Specifically, warbler NFC detections increased sharply in the first few hours after civil sunset and peaked before the middle of the night, then decreased more slowly through civil sunrise. The primary discrepancy among locations was the relatively reduced warbler NFC detections in the last quarter of the night prior to civil sunrise at southern Block Island sites (Figure 4D). Additionally, the non-zero density of NFC detections at civil sunset suggests warbler migration was underway by this time (Figure 4). Compared to warblers, sparrow NFC detections increased more slowly after civil sunset and exhibited a more protracted period of peak activity centered around the middle of the night, roughly 2330 h EST (Figure 4). Again southern Block Island sites were the exception to this general pattern, as sparrow NFC detections was distinctly reduced near civil sunset and sunrise, producing a more pronounced peak of activity near the middle of the night (Figure 4D).

**Figure 5:**
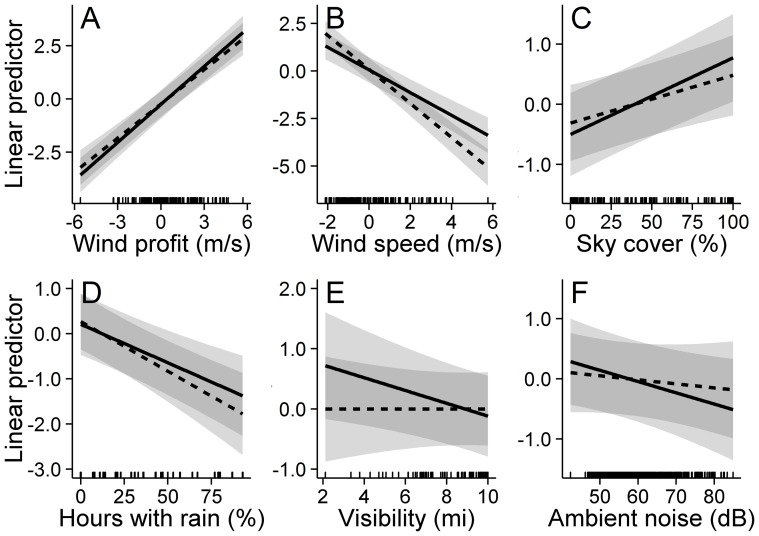
Warbler and sparrow flight call relationships with atmospheric and ambient conditions. Changes in warbler (solid line) and sparrow (dashed line) nocturnal flight call (NFC) detections during the 2010 and 2011 fall migrations as a function of average regional atmospheric conditions (A–E) and ambient noise (F); associations were estimated with generalized additive models. We illustrate each variable's association (and 95% confidence interval) with the linear predictor of NFC detections when all other variables are at their mean value; we excluded the intercept and site-specific effects from the linear predictor to facilitate the comparison of effect magnitudes among variables. Note that each unit change in the linear predictor represents nearly a tripling of NFC detections. Rug plots illustrate the distribution of the input variables.

Warbler and sparrow NFC detections increased substantially with wind conditions indicative of recent cold front passage and favorable for coastal migration (Table 3, Figure 5A). Additionally, warbler and sparrow NFC detections decreased with an increasing regional presence of rain (Table 3, Figure 5D). Warbler and sparrow NFC detections increased under cloudier skies (and lower cloud ceilings; Table 3, Figure 5C), but decreased visibility was associated with increased detections only in warblers (Table 3, Figure 5E). NFC detections decreased considerably with increasing wind speeds (Table 3, Figure 5B). Ambient noise only marginally decreased the detection of warbler flight calls and exhibited little association with the detection of sparrow NFCs (Table 3, Figure 5F).

**Table 3:** Relationships between nightly warbler and sparrow nocturnal flight call (NFC) detections and average regional nightly atmospheric or ambient noise conditions estimated via generalized additive models.

		Warblers			Sparrows		
Variable^a^	Expected association	Estimate (SE)	*t* b	*P* c	Estimate (SE)	*t* b	*P* c
Wind profit	+	1.13 (0.08)	14.99	<0.001	1.01 (0.09)	11.31	<0.001
Wind speed	−	−0.91 (0.09)	−10.24	<0.001	−1.37 (0.10)	−13.57	<0.001
Rain	−	−0.37 (0.09)	−4.08	<0.001	−0.47 (0.10)	−4.63	<0.001
Cloud cover	+	0.40 (0.08)	4.77	<0.001	0.24 (0.09)	2.78	0.006
Visibility	−	−0.19 (0.07)	−2.66	0.008	0.00 (0.08)	0.03	0.97
Noise	−	−0.16 (0.08)	−2.11	0.035	−0.07 (0.08)	−0.81	0.42

a Input variables were centered and scaled; thus, exponentiation of parameter estimates provides the average change in NFC detections per standard deviation change of the input variable. Standard deviations of input variables: wind profit (1.91 m/s), wind speed (1.53 m/s), rain (22.57%), cloud cover (35.27%), visibility (1.70 mi), noise (8.28 dB).

b 603 residual degrees of freedom.

c Although the expected associations are one-directional, we report *P* from the two-sided test to avoid missing large differences in the unexpected direction [72].

We found little evidence that the efficiency of our automated detector algorithm varied among sites (likelihood ratio test [LRT]: χ^2^ = 9.92, df = 7, P = 0.19) or with background noise in relatively low-noise conditions (LRT: χ^2^ = 0.03, df = 1, P = 0.86). The automated detector extracted 55–60% (95% confidence interval) of flight calls attributable to warblers and sparrows under reasonably good recording conditions.

## Discussion

We used NFC recordings of at least 22 migratory songbirds (14 warbler and 8 sparrow species) during fall migration from multiple sites along mainland and island coasts of Rhode Island to evaluate hypotheses regarding NFC detections. Patterns of warbler and sparrow NFC detections largely supported our expectations that (1) NFC detections were associated positively and strongly with wind profit and negatively with regional precipitation; (2) NFCs increased with reduced visibility for migrants (e.g., increased cloud cover); (3) NFCs decreased with higher wind speeds, presumably due mostly to increased ambient noise; and (4) coastal mainland sites recorded five to nine times more NFCs, on average, than coastal nearshore or offshore island sites. However, we found little evidence that (5) nightly or intra-night patterns of NFCs reflected the well-documented latitudinal patterns of migrant abundance on Block Island.

### Associations of NFC detectability with atmospheric conditions

Atmospheric and ambient conditions can influence the detection rates of NFCs directly by inducing a change in the rate at which migrants call, or indirectly by influencing the number of birds aloft or NFC detectability. Certain atmospheric conditions commonly are associated with increased numbers of birds aloft in north temperate areas (e.g., front passage, wind conditions, and precipitation; [1], [2], [4]) and increase the likelihood of migrant concentrations along the Atlantic Coast of North America [22], [28]. We formulated wind profit to reflect wind conditions favorable for migration in general (i.e., the northerly component that typically follows cold front passage and building high pressure) but also to favor a westerly component more likely to induce a flight path towards the coast and offshore displacement. While northeast and east winds may provide favorable tailwinds to migrating songbirds in our region [2], [4], we expected winds from these directions to diminish the concentrating influence of the Atlantic Coast on southbound migrant activity. Consequently, our formulation of wind profit ascribed reduced or negative values to these wind conditions. We suggest the strong association of warbler and sparrow NFC detections with wind profit supports the idea that NFC detections generally reflect the number of birds aloft. We note that in addition to wind profit, several alternatives exist for capturing the multivariate problem of wind assistance, each making different assumptions regarding the behavior of the organism of interest [46].

Unlike wind profit, the extent to which other atmospheric conditions influence NFC detections directly or indirectly is less clear and not likely to be mutually exclusive. Therefore, atmospheric conditions likely complicate the relationship between the number of vocal birds aloft and NFC detections. For example, precipitation is thought to suppress migration [1], [2], [4], which concurs with the negative association of regional precipitation with NFC detections we documented in this study. However, consistent precipitation also compromises the detectability of NFCs by increasing the background noise (see below). In contrast, light to moderate precipitation may decrease visibility without hindering migration and thus induce increased calling rates [35], [47] or lower flight altitudes [8], thereby increasing detections.

Cloud cover and visibility describe additional conditions under which the influence on NFC detections may be direct or indirect. NFC detections are known to be positively related to increasing cloud cover (or decreasing cloud ceiling, which correlated very strongly with cloud cover in this study) or decreasing visibility (reviewed in [35]; see also [47]). Farnsworth [35] suggested that increased calling rates by individuals under conditions of poor visibility may be adaptive for maintaining contact, avoiding collisions, and coordinating migratory behavior, particularly in inexperienced migrants [48]; this hypothesis implies a behavior-modifying influence on calling rates. Its potential pertinence to inexperienced migrants is particularly relevant to this study, as the vast majority of autumnal songbird migrants along the coast are young birds performing their first migration [49]–[53]. But indirect effects seem equally plausible; poor visibility, cloud cover, and low cloud ceiling may decrease flight altitudes relative to clearer nights, placing more migrants within NFC detection range. Although we documented the expected increase in NFC detections with increasing cloud cover (warblers and sparrows) and decreasing visibility (warblers only), we were unable to distinguish between changes in calling or flight behaviors.

We further expected certain atmospheric and biological conditions to increase background noise and decrease our ability to detect NFCs, and thus associate negatively to NFC detections. Wind and insects comprised the primary sources of noise in this study. Wind speed associated strongly and negatively with NFC detections. The strong correlation between wind speed and background noise measurements at each site (r = 0.59, P<0.001, df = 622; ‘within-site’ correlation *sensu* [57]) suggests wind noise importantly reduced the detection of NFCs. Nonetheless, higher wind speeds might also decrease the number of migrants aloft, particularly when opposing the direction of travel [4], [54], although high wind conditions may also result in lower flight altitudes [54]–[56] and thus possibly increase detectability. We suggest that the negative effect of wind speed reflected primarily an increase in background noise and an associated decrease in the detectability of NFCs more so than a decrease in warbler and sparrow abundance aloft [34]. Insect noise was the primary non-wind source of background noise above 6 kHz. Our manual detector evaluation revealed a clear reduction in NFC detection due to insect noise in some instances (A. D. Smith unpubl. data), but its irregular and ephemeral occurrence made it impractical to quantify on a nightly basis. Insect noise occurred less commonly as the season progressed. While high background noise levels universally impaired NFC detections, intermediate noise levels may have disproportionately discriminated against species that produce calls not contained completely in the frequency range of our detector or less powerful calls, or that fly at higher altitudes. For example, most warbler NFCs occur above 6 kHz, but a few common species produce flight calls that regularly drop below 6 kHz (Table 2). We suggest that these calls were more sensitive to noise levels given our detector settings. In contrast, the NFCs of all sparrow species in this study occur entirely above 6 kHz [30]. This perhaps explains the weak influence of non-wind ambient noise on warbler NFC detection and the apparent lack of such an influence for sparrows.

### NFC detections and coastal context

We recorded warbler and sparrow NFCs in multiple coastal contexts along the Atlantic Coast of the northeastern United States, an important migratory corridor during autumn migration. NFC detections occurred episodically over the fall migration season, similar to migration intensity in the region (e.g., [9], [15], [17], [58]), presumably the result of most migrants coinciding movements with ephemerally favorable conditions [2], [4], [28], [39], [59]. Although offshore islands often offer excellent opportunities to observe high densities of migrants [20], [37], we expected that most migrants would move over land or near the coast, rather than offshore, and thus would detect more NFCs at mainland sites compared to offshore sites. Indeed, warbler and sparrow NFC detections were considerably higher at our two mainland sites. Radar studies suggest the difference in NFC detections between coastal contexts along the Atlantic Coast reflects migrant abundance rather than a difference in the calling behavior or flight altitudes of birds, as the bulk of migration intensity occurs along the coast and inland, not over ocean, excluding water crossings from Nova Scotia over the Gulf of Maine [15]–[17], [60]. Furthermore, if patterns of NFC detections reflected changes in calling behavior more so than abundance, we might reasonably expect the opposite pattern (i.e., birds displaced offshore increase calling rates). Flight altitude might play some role in the observed pattern, but the data are scarce and mixed and often complicated by radar peculiarities (see, e.g., [8], [21], [25]).

There exists a well-documented pattern in ‘on-the-ground’ migrant densities on Block Island: migrants occur in higher densities on the northern half of the island, where they prepare for reoriented flights to the mainland or subsequent migratory flights [20], [37]. Indeed, two migration banding operations on the island ([52]; USFWS unpubl. data) exploit the phenomenon, as have multiple previous studies [61]–[65]. We thus expected a similar latitudinal pattern in NFC detections among Block Island sites; however, we found little evidence for this pattern (or differences among sites in general), suggesting that concentrations of migrants on the northern half of Block Island result primarily from redistribution after landfall (e.g., [22], [24], [66], [67]). Indeed, regular observation of significant diurnal, northerly movements of migrants on offshore islands following nights of active southerly migration provide evidence of such a redistribution ([20], [37]; A. D. Smith pers. obs.).

Migration activity along the Atlantic Coast, as assessed by radar, generally peaks in the few hours following sunset and declines steadily thereafter [9], [33], [58], [60], [68]. Comparisons of NFC detections with radar are few but suggest that NFC detections follows a similar pattern ([34]; but see the New York data in [33]) or peak up to a few hours later, usually near or just after the middle of the night [33], [69]. Seasonal patterns of intra-night NFC detections in this study (Figure 4) support an apparent delay in peak NFC detections relative to expectations from previous radar work. The patterns also suggest that sparrows migrate, or at least call, slightly later in the night on average than warblers (Figure 4). The intra-night patterns of warbler and sparrow NFC detections were generally consistent for the larger coastal context (i.e., mainland vs. island) and among latitudinal contexts on Block Island, with the possible exception of sparrow NFC detections at southern Block Island sites (Figure 4D). For reasons that remain unclear, the reduced activity near sunrise and sunset at these sites suggests that fewer sparrows are landing and settling on southern Block Island. Finally, rather than comparing seasonal averages of intra-night activity, more detailed work will be necessary to evaluate the variability of the relationship between migrant density and concurrent NFC detections (e.g., [34]).

### NFC species composition

Acoustic monitoring is relatively inexpensive compared to radar, the equipment can be automated, and it provides information not readily obtained from other methodologies, including species composition and phenology information for vocal species and the ability to detect secretive, rare, or species otherwise difficult to survey [70], [71]. However, inferring the relative abundances of calling species using patterns of NFCs is complicated because several common species do not regularly vocalize during migration (e.g., flycatchers, vireos, mimids; [30], [35], [36]) whereas other species regularly vocalize during migration and so may be over-represented in NFC recordings (e.g., Savannah Sparrows; [71]). For example, a comparison of NFC detections at four of our microphone locations with capture rates at active and close (<500 m) banding operation suggests that Savannah and Chipping Sparrows were likely over-represented in NFC recordings whereas Yellow-rumped Warblers were likely under-represented in NFC recordings. Clearly, inferring the relative abundances of calling species using patterns of NFCs requires more knowledge of calling rates among species [36] and species-specific differences in NFC detection. Despite these potential complications in interpreting NFC data and inferring migration intensity, the acoustic monitoring of NFCs provides a viable and complementary methodology for exploring the spatiotemporal patterns of songbird migration (e.g., [29], [71]; see also oldbird.org), as well as evaluating the atmospheric conditions that shape these patterns.
